# Accuracy of Patient-Specific Drilling Guides in Acetabular Fracture Surgery: A Human Cadaver Study

**DOI:** 10.3390/jpm11080763

**Published:** 2021-08-03

**Authors:** Anne M. L. Meesters, Nick Assink, Kaj ten Duis, Eelco M. Fennema, Joep Kraeima, Max J. H. Witjes, Jean-Paul P. M. de Vries, Vincent M. A. Stirler, Frank F. A. IJpma

**Affiliations:** 1 Department of Surgery, University Medical Centre Groningen, University of Groningen, 9713 GZ Groningen, The Netherlands; a.m.l.meesters@umcg.nl (A.M.L.M.); n.assink@umcg.nl (N.A.); k.ten.duis@umcg.nl (K.t.D.); emfennema@gmail.com (E.M.F.); j.p.p.m.de.vries@umcg.nl (J.-P.P.M.d.V.); vincent.stirler@gmail.com (V.M.A.S.); 2 3D Lab, University Medical Centre Groningen, University of Groningen, 9713 GZ Groningen, The Netherlands; j.kraeima@umcg.nl (J.K.); m.j.h.witjes@umcg.nl (M.J.H.W.); 3 Department of Oral and Maxillofacial Surgery, University Medical Centre Groningen, University of Groningen, 9713 GZ Groningen, The Netherlands

**Keywords:** 3D print, 3D virtual surgical planning, acetabular fracture, patient-specific, surgical guides

## Abstract

Due to the complex anatomical shape of the pelvis, screw placement can be challenging in acetabular fracture surgery. This study aims to assess the accuracy of screw placement using patient-specific surgical drilling guides applied to pre-contoured conventional implants in acetabular fracture surgery. CT scans were made of four human cadavers to create 3D models of each (unfractured) pelvis. Implants were pre-contoured on 3D printed pelvic models and optically scanned. Following virtual preoperative planning, surgical drilling guides were designed to fit on top of the implant and were 3D printed. The differences between the pre-planned and actual screw directions (degrees) and screw entry points (mm) were assessed from the pre- and postoperative CT-scans. The median difference between the planned and actual screw direction was 5.9° (IQR: 4–8°) for the in-plate screws and 7.6° (IQR: 6–10°) for the infra-acetabular and column screws. The median entry point differences were 3.6 (IQR: 2–5) mm for the in-plate screws and 2.6 (IQR: 2–3) mm for the infra-acetabular and column screws. No screws penetrated into the hip joint or caused soft tissue injuries. Three-dimensional preoperative planning in combination with surgical guides that envelope pre-contoured conventional implants result in accurate screw placement during acetabular fracture surgery.

## 1. Introduction

The implants currently used in acetabular fracture surgery often require multiple bending and contouring manoeuvres intraoperatively, which may result in malpositioning, suboptimal fitting, longer operation time, and therewith increased risk of infection [1]. In addition, it may be hard to determine and verify the optimal screw positions using fluoroscopy, due to the complex geometry of the pelvis [2]. Good cortical grip strength requires optimal screw entry points and directions. Besides the in-plate screws, some additional infra-acetabular (Pohlemann screw [3]) and column screws might be required to increase the fixation strength [2,4,5,6,7,8,9,10]. Those screws can be difficult to aim due to narrow bone corridors, differences in pelvic geometry between individuals, soft tissue hindrances, a steep slope at the entry point, and difficulties in verifying the screw positions using fluoroscopy [2,6,7,8,9,11].

Over the last few years, innovative 3D technologies have been increasingly used for the surgical treatment of pelvic fractures. Virtual 3D models could aid in understanding complex fracture patterns better, facilitating treatment decisions, as well as for printing tangible 3D models, which could be used for pre-contouring and fitting osteosynthesis plates [12,13,14,15]. Moreover, 3D surgical planning could aid in predetermining accurate screws positions [16]. The clinical application of these 3D technologies may contribute to shorter operation times, reduced blood loss, improved implant and screw positions, and less residual displacement of the fracture fragments [17,18,19,20,21,22]. Recently, we developed an innovative workflow for the clinical application of patient-specific osteosynthesis plates with drilling guides in acetabular fracture surgery [23]. This personalized surgical approach has enabled the execution of the preoperative plan and to attain the predetermined osteosynthesis plate and screw positions.

We hypothesized that 3D surgical planning and drilling guides can help in positioning commercially available implants and in aiming in-plate, infra-acetabular, and column screws in the pre-planned directions. Therefore, the aim of this human cadaver study was to assess screw placement accuracy on applying patient-specific surgical drilling guides as an adjunct to conventional implants in acetabular fracture surgery.

## 2. Materials and Methods

### 2.1. Specimens

Four full-body Thiel-embalmed human cadavers (three males and one female) were obtained from the anatomy department [24]. A pelvic CT scan was made of each cadaver according to our standard imaging protocol (0.6 mm slice thickness, voxel size 0.4 mm).

### 2.2. 3D Surgical Planning

The Mimics Medical 19.0 software (Materialise, Leuven, Belgium) was used to generate a 3D model of the pelvis from the CT data. A pre-set bone threshold (226–3071 Hounsfield Units) was applied and the pelvic bone was digitally separated from the sacrum and femur using the split mask tool. Next, the areas of the pelvis where the implants would be placed were virtually separated from the pelvis and 3D printed out of Poly Lactic Acid using a fused deposition modelling (FDM, Ultimaker, Utrecht, The Netherlands) printer. Conventional implants were manually contoured from these 3D prints, namely 9- or 10-hole straight plates (DePuy Synthes, Raynham, MA, USA) for posterior fixation, and suprapectineal plates (Stryker, Kalamazoo, MI, USA) for anterior fixation.

**Figure 1:**
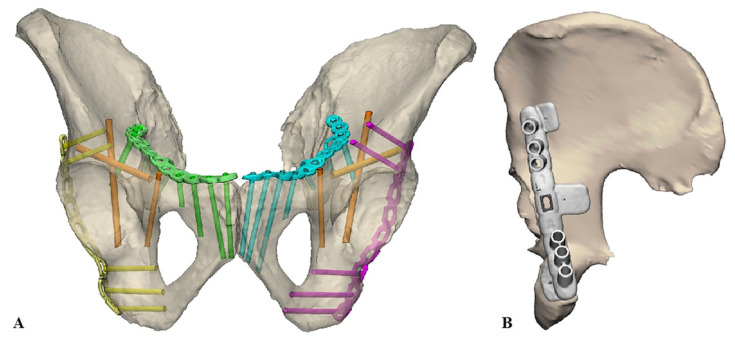
3D preoperative planning. (**A**) Overview of the pre-planned screw directions and the pre-contoured implants on the pelvis. The cylinders represent the pre-planned screw directions of the in-plate screws (yellow, green, blue, and purple) and in orange the infra-acetabular and column screws. (**B**) Pre-contoured posterior implant and the surgical guide with the bone extensions.

**Figure 2:**
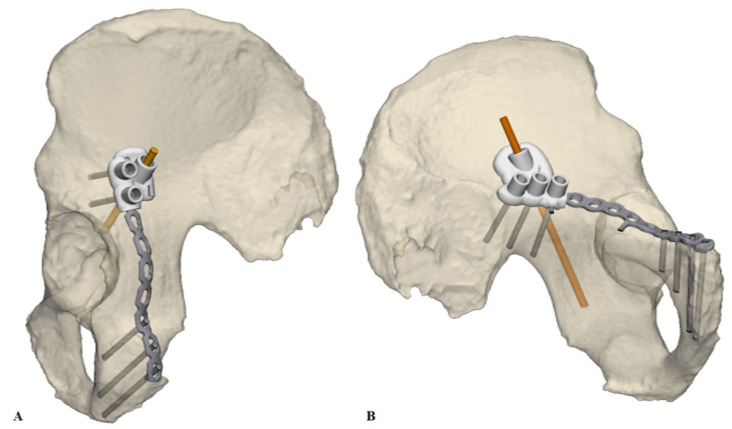
Drilling guides. Separate drilling guides were designed for the anterior and posterior column screws. (**A**) The drilling guide for the retrograde anterior column screw, (**B**) The drilling guide for the posterior column screw.

**Figure 3:**
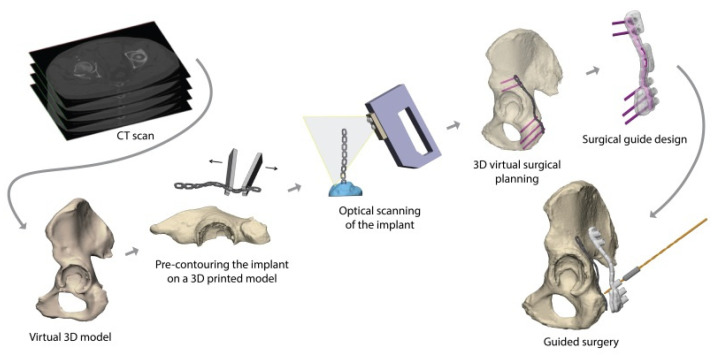
Workflow of the preoperative planning. A 3D model is created from the CT scan. The implant was pre-contoured on a 3D printed model and optically scanned. The implant was matched with the 3D model, and the screw directions and positions were predetermined. The personalized surgical drilling guide was designed and 3D printed for intraoperative use.

After pre-contouring, the implants were scanned optically with an Artec Space Spider (Artec 3D, Luxembourg, Luxembourg) to generate a Stereolithography (STL) model. In addition, the implants were optically scanned on top of the 3D print, which was used to position the pre-contoured implants virtually on the pelvic models. Afterwards, the optimal screw trajectories were pre-determined in the 3-Matic 11.0 (Materialise, Leuven, Belgium) software (Figure 1A). The entry point of the infra-acetabular, or Pohlemann screw, was virtually projected through the fifth anterior hole of the suprapectineal plate, if allowed by the shape of the pelvis. Posterior column screws and retrograde anterior column screws were included in the preoperative planning. All the screws were 3.5 mm in diameter, except for the column screws, which had a diameter of 4.5 mm. Case-specific surgical drilling guides were designed to translate the virtual planning to the surgical procedure. The drilling guides, which were constructed to fit on top of the implant, assisted the surgeon to aim the screws in the pre-planned direction. Furthermore, the guides were equipped with several supporting extensions, including k-wire holes, to direct the implant onto the pre-planned position on the bone (Figure 1B). The drilling guides consisted of multiple cylinders (8 mm in height) in which a stainless-steel drill sleeve (316 L, 25 mm in length, with a diameter of 2.6 mm for a 2.5 mm drill) could be inserted to guide the drill and place the screws in the pre-planned direction. For the anterior (Figure 2A) and posterior column screws (Figure 2B), separate drilling guides were designed. The drilling guides were 3D laser-printed from polyamide powder. Figure 3 provides an overview of the workflow.

### 2.3. Surgical Procedure

**Figure 4:**
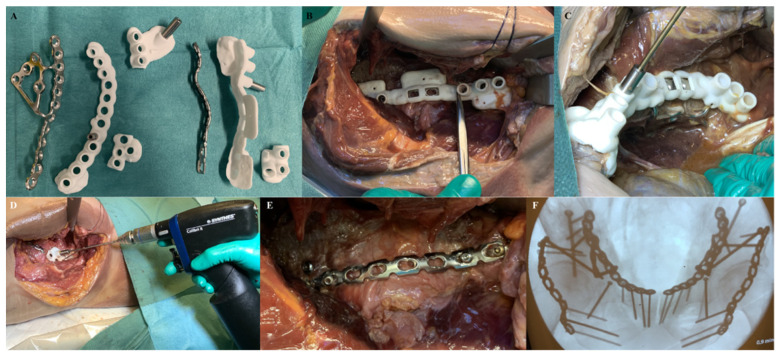
**Surgery.** (**A**) The pre-contoured implants, surgical guides including the extensions and the drill sleeves. (**B**) Perioperative view through a Kocher-Langenbeck approach with a clear view on a posterior plate with surgical guide. (**C**) Perioperative view through a modified Stoppa approach with a clear view on the suprapectineal plate with surgical guide, drill sleeve, and drill. (**D**) Fixation of the posterior plate by using a separate surgical guide with the drill sleeve and drill. (**E**) Posterior plate fixated to the pelvis. (**F**) Intraoperative fluoroscopy at the end of the surgical procedure.

In each cadaver, both the Kocher-Langenbeck and the anterior intrapelvic approaches were performed by two pelvic surgeons. These approaches were extended with an additional lateral window to place the posterior column screws. The pelvis was exposed according to the standard of care. After this, the drilling guide (Figure 4A) was placed on top of the implant and both were positioned on the bone (Figure 4B,C). The bone supporting extensions of the drilling guides enabled visual and haptic feedback for correct implant positioning, which was verified using intraoperative fluoroscopy. A drill sleeve was inserted into the guide’s cylinders to aim the drill in the pre-planned direction (Figure 4C,D). After drilling, the drill sleeve was removed, and a screw was inserted. The drill guide was removed from the implant before skin closure (Figure 4E). Finally, the implant and screw positions were verified with intraoperative fluoroscopy, and the wounds were closed (Figure 4F).

### 2.4. Postoperative Evaluation–Accuracy of Guided Screw Insertion

**Figure 5:**
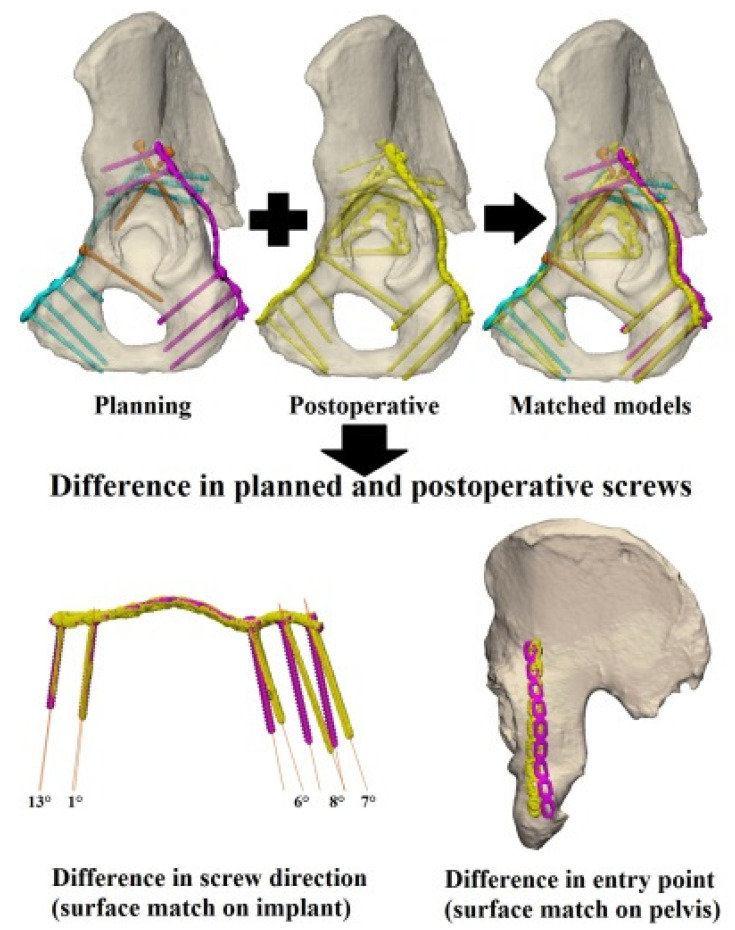
Postoperative analysis. The planned screw directions and positions (pink for posterior, blue for anterior, and orange for the infra-acetabular and column screws) are matched with the postoperative actual screw directions and positions (in yellow). The difference in the screw direction (lower left) and screw entry point (lower right) were measured.

In each case, a standard pelvic CT scan (0.6 mm slice thickness; iterative metal artefact reduction) was made postoperatively in order to evaluate the accuracy of the screw directions and the screw positions of the implant. The CT data was used to generate a 3D model of the pelvis with the implants and screws in situ. The postoperative 3D model was surface-based matched with the preoperative 3D model of the pelvic bone, in order to assess the differences between the preoperative screw entry points (in mm) and the postoperative results (Figure 5). The screw direction differences were assessed by comparing the pre- and postoperative screw trajectories, through matching the postoperative implant with the preoperative implant. The 3D deviation was measured between the pre- and postoperative screw directions in degrees, with the screw heads as reference points and using a Cartesian coordinate system. 

## 3. Results

### Postoperative Evaluation—Accuracy of Guided Screw Insertion

Four bilateral anterior intrapelvic approaches and four bilateral Kocher-Langenbeck approaches were performed on four human cadavers. A total of 8 suprapectineal plates with 54 in-plate screws and 8 posterior plates with 43 in-plate screws were placed. Moreover, a total of six infra-acetabular screws, four anterior column screws, and four posterior column screws were placed. Thus, a total of 111 screws were placed with the help of the surgical drilling guides. None of the implants needed additional bending during the surgery and no revisions had to be made after checking with fluoroscopy.

The median difference between the planned and actual direction of all the screws was 6.1 (IQR: 4–8) degrees. The median difference in the direction of the in-plate screws was 5.9 (IQR: 4–8) degrees (posterior screws: 5.2 degrees; anterior screws: 6.1 degrees) (Table 1). The difference in the direction of the in-plate screws for each session can be found in the Supplementary Materials Figures S1–S4. Moreover, the median difference between the planned and executed screw direction was 7.6 (IQR: 6–10) degrees for the infra-acetabular and column screws (anterior column screws: 4.1 degrees; infra-acetabular screws: 7.5 degrees; posterior column screws: 9.6 degrees) (Table 2).

The median difference between the planned and actual entry point of the screws was 3.4 (IQR: 2–4) mm. The median difference of the in-plate screw entry points was 3.6 (IQR: 2–5) mm (posterior screws: 2.9 mm; anterior screws: 4.4 mm). The median difference of the infra-acetabular and column screw entry points was 2.6 (IQR: 2–3) mm. Additionally, the smallest median difference in entry point was seen with the infra-acetabular and column screws (2.6 mm). None of the screws penetrated into the hip joint or caused soft tissue injuries.

## 4. Discussion

The purpose of this study, which was to assess screw placement accuracy during acetabular fracture surgery on using patient-specific surgical drilling guides as an add-on to pre-contoured conventional implants, was fulfilled. The personalized surgical drilling guides translated the virtual planning accurately to the surgical procedure by helping the surgeon steer the screws in the pre-planned direction, with only a median difference in screw direction of 6.1 degrees and a median difference in screw entry point of 3.4 mm between the planning and the actual operative result.

Regarding acetabular fractures, surgical guides have only been used in patients in combination with patient-specific implants so far by our group, which resulted in an 7.1 degree difference in screw direction between the planning and actual surgery [23]. This is in line with the 6.1 degrees in our current study. A margin of 6.1-degree difference between the preoperative planning and execution is acceptable for clinical use, because this margin can be incorporated in the virtual surgical planning. Moreover, none of the screws penetrated into the hip joint or caused soft tissue injuries. Two studies investigated the use of surgical guides in the pelvis of human cadavers, which resulted in accurate screw placement [25,26]. However, both their technique and measurement method were considerably different from ours, e.g., they did not report on differences between planned and actual screw directions, which made comparing the results challenging. Huang et al. used patient-specific surgical guides in combination with conventional implants for tibial plateau fractures [27]. Their results are similar to ours (6.3 degrees difference and 0.2 to 0.8 mm difference in the *x*-, *y*-, and *z*-axes). However, it should be noted that they measured their differences in three different planes whereas our differences were not limited to separate planes. Another application of surgical drilling guides, for pedicle and lateral mass screws of the spine, resulted in higher accuracy compared to our results (0.8 mm difference in entry point and 3.2 degrees difference in direction between planning and actual screw placement) [28]. A possible explanation for this can be that the spinous process has more anatomical reference points to serve as a stable base for the guide compared to the sloping surface of the pelvis. Moreover, soft tissues can hamper the direction of the drill more easily in pelvic surgery compared to spinal surgery.

The surgical guides were easy to use, and the screws could be placed without any difficulties. This resulted in accurate screw placement but, as expected, some small but clinically acceptable inaccuracies remained when translating the virtual surgical plan to the surgery itself. One of the causes is the sloping surface of the pelvis. Another reason is the lack of reference points in the limited surgical field for exact positioning of the implant. In addition, on fixating the implant on the bone, the implant deforms slightly to the shape of the pelvis. Subsequently, this will result in a slight deviation from the planning when the surgical guide is positioned on top of the implant, but the guide still had a good fit on top of the implant. The 3D models were based on CT scans with a small slice thickness and voxel size. Moreover, validated and certified software was used by experienced observers, with a standardized threshold for bone, thus the influence of the 3D models on the accuracy was minimal. The posterior column screws resulted in greater differences between the planned and actual screw directions (9.6 degrees median difference). This was suspected to be mainly caused by the soft tissue interference. Furthermore, the use of a separate drilling guide to place the infra-acetabular screws resulted in similar differences between the planned and actual screw directions and entry points.

We show that our method is feasible for future clinical settings. 3D technology enables the surgeon to plan the surgery more precisely beforehand, to determine the screw positions and directions, and to discuss the virtual surgical plan with colleagues before the actual surgery. However, the preoperative planning and guide design is costly in terms of time and should therefore be well evaluated before widespread use. When implementing this workflow, one needs to consider the available time from patient admission to surgery. On average, it took us six days from the preoperative CT scan until the surgical guides were ready for surgery. The most time-consuming part of our study was the 3D printing and the delivery of the surgical guides by the manufacturer. Nonetheless, if on-site printing were possible, the guides would be ready in five days instead of six days. Another potential benefit might be a reduction in operating time since the plate is already pre-contoured and the screw trajectories are already predetermined. Additionally, fluoroscopic verification of the implant and screw positions can be minimized by using surgical guides, especially for the infra-acetabular and column screws, which could lead to less radiation exposure.

A major strength of our study is that the 3D virtual surgical planning was executed on human pelvises and the surgeries were performed by two pelvic surgeons with >5 years of experience. The implants and screws are used widely in clinical practice. In addition, a validated and certified software was used for the preoperative planning. A limitation of this study is that it is an experimental design with a limited number of human cadavers. Moreover, no control group was used, because we know from clinical practise that freehand screw placement is challenging in acetabular fracture surgery. The present study is considered a feasibility study aiming to assess the accuracy of ‘controlled’ screw placement by using surgical guides. Furthermore, no fractures were present in the human cadavers, because that is hard to realise in an experimental setting. However, a recent study using surgical guides with patient-specific implants demonstrated similar results regarding screw placement accuracy as the present study [23]. In addition, a virtually reduced pelvis or the contralateral mirrored hemipelvis can be 3D printed for pre-contouring the implant, because the shape of the left and right hemipelvis are almost identical [29]. One could virtually reduce the fracture [23], using a statistical shape model as a template [30,31], when encountering contralateral acetabular fractures or associate pelvic ring injuries. Although 3D virtual surgical planning can be time-consuming, and it is not widely available yet, surgical guides should be considered as an adjunct to conventional implants, thus making it potentially widely applicable in other hospitals.

In conclusion, 3D virtual surgical planning and patient-specific drilling guides can be used as an adjunct to conventional implants in acetabular fracture surgery, to translate the virtual surgical planning to the operation. The use of patient-specific guides allows for accurate implant positioning and accurate placement of in-plate, infra-acetabular, and column screws. Overall, the preoperative planning could be accurately executed during guided surgery. The small clinically acceptable inaccuracies in screw direction and entry point between the preoperative planning and the actual operative result could be explained by the sloping surface of the pelvis and the lack of reference points in the limited surgical field for exact positioning of the implant.

## Figures and Tables

**Table 1:** In-plate screw difference. The number of screws (N), the median difference in entry point (∆ position) and the median difference in direction (∆ direction) between the preoperative and postoperative 3D model of the in-plate screws for each session with the interquartile range (IQR).

	Anterior In-Plate Screws			Posterior In-Plate Screws		
Case	N	∆ Entry Point (IQR)	∆ Direction (IQR)	N	∆ Entry Point (IQR)	∆ Direction (IQR)
1	16	3.6 (3–4) mm	5.0 (3–7)°	11	7.1 (5–10) mm	3.3 (3–5)°
2	16	2.1 (2–3) mm	6.3 (5–8)°	10	3.6 (3–4) mm	5.4 (4–8)°
3	8	2.5 (2–3) mm	5.0 (4–6)°	12	5.2 (4–6) mm	6.3 (5–7)°
4	14	3.0 (2–3) mm	8.2 (5–9)°	10	3.9 (4–5) mm	4.5 (4–7)°
Median (IQR)		2.9 (2–4) mm	6.1 (4–9)°		4.4 (3–6) mm	5.2 (3–7)°

**Table 2:** Column and infra-acetabular screw difference. The difference in entry point (∆ P) and direction (∆ D) of the infra-acetabular, anterior column, and posterior column screws between the preoperative and postoperative 3D model for each case. Finally, the median differences with the interquartile range (IQR) are presented for each type of screw.

	Infra-Acetabular Screws		Anterior Column Screws		Posterior Column Screws	
Case	∆ P	∆ D	∆ P	∆ D	∆ P	∆ D
1	3.0 mm	8.4°	N/A	N/A	N/A	N/A
	4.0 mm	6.3°				
2	1.7 mm	6.6°	N/A	N/A	N/A	N/A
	2.1 mm	8.9°				
3	N/A	N/A	3.6 mm	10.6°	3.0 mm	8.9°
			1.0 mm	1.4°	1.0 mm	10.2°
4	3.3 mm	11.8°	7.0 mm	2.4°	2.2 mm	6.7°
	1.5 mm	4.1°	2.1 mm	5.7°	12.4 mm	16.5°
Median (IQR)	2.6 (2–3) mm	7.5 (6–9)°	2.8 (2–4) mm	4.1 (2–7)°	2.6 (2–5) mm	9.6 (8–12)°

N/A: Not applicable.

## Data Availability

The authors declare that the data supporting the findings of this study are available within the paper.

## Supplementary Materials

The following are available online at https://www.mdpi.com/article/10.3390/jpm11080763/s1, Figure S1: Difference in screw direction for session one, Figure S2: Difference in screw direction for session two, Figure S3: Difference in screw direction for session three, Figure S4: Difference in screw direction for session four.
