# Supplementary figures and images for: Accuracy of Patient-Specific Drilling Guides in Acetabular Fracture Surgery: A Human Cadaver Study
Source: J Pers Med. 2021 Aug 3;11(8):763. doi: 10.3390/jpm11080763 (PMC8400721; doi:10.3390/jpm11080763)

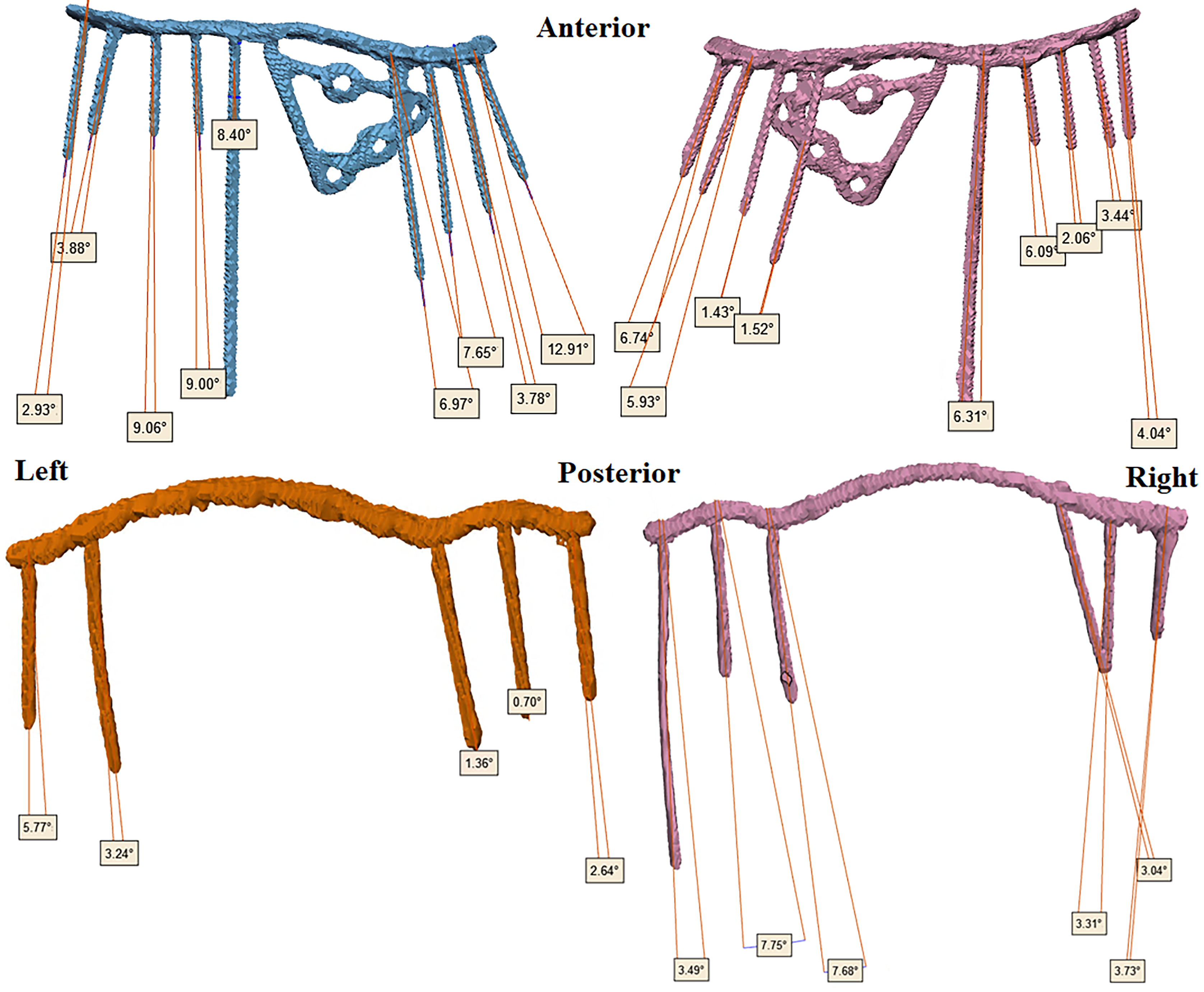

Supplement: Supplementary file 1 [file jpm-11-00763-s001.zip › Figure S1.png]

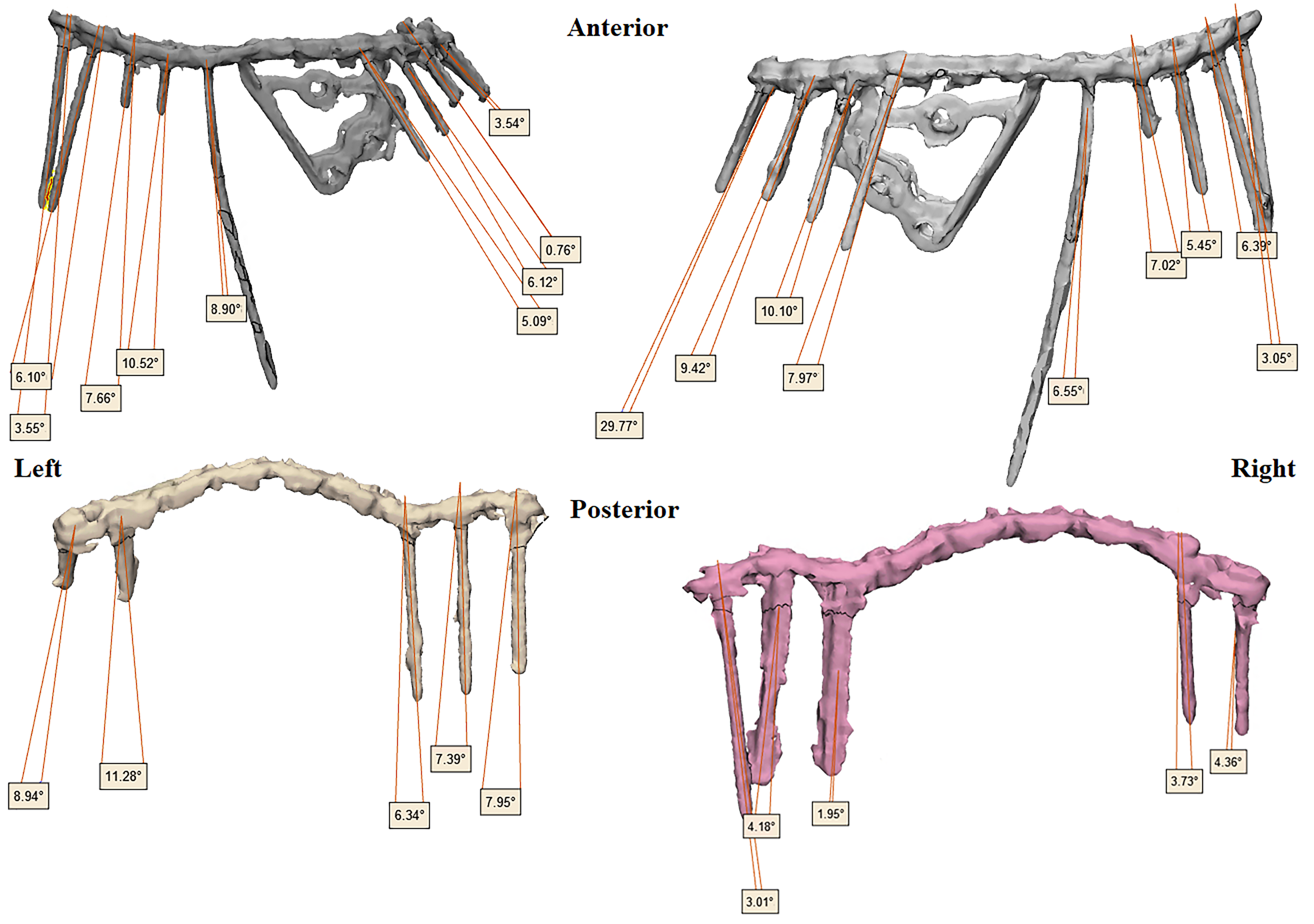

Supplement: Supplementary file 1 [file jpm-11-00763-s001.zip › Figure S2.png]

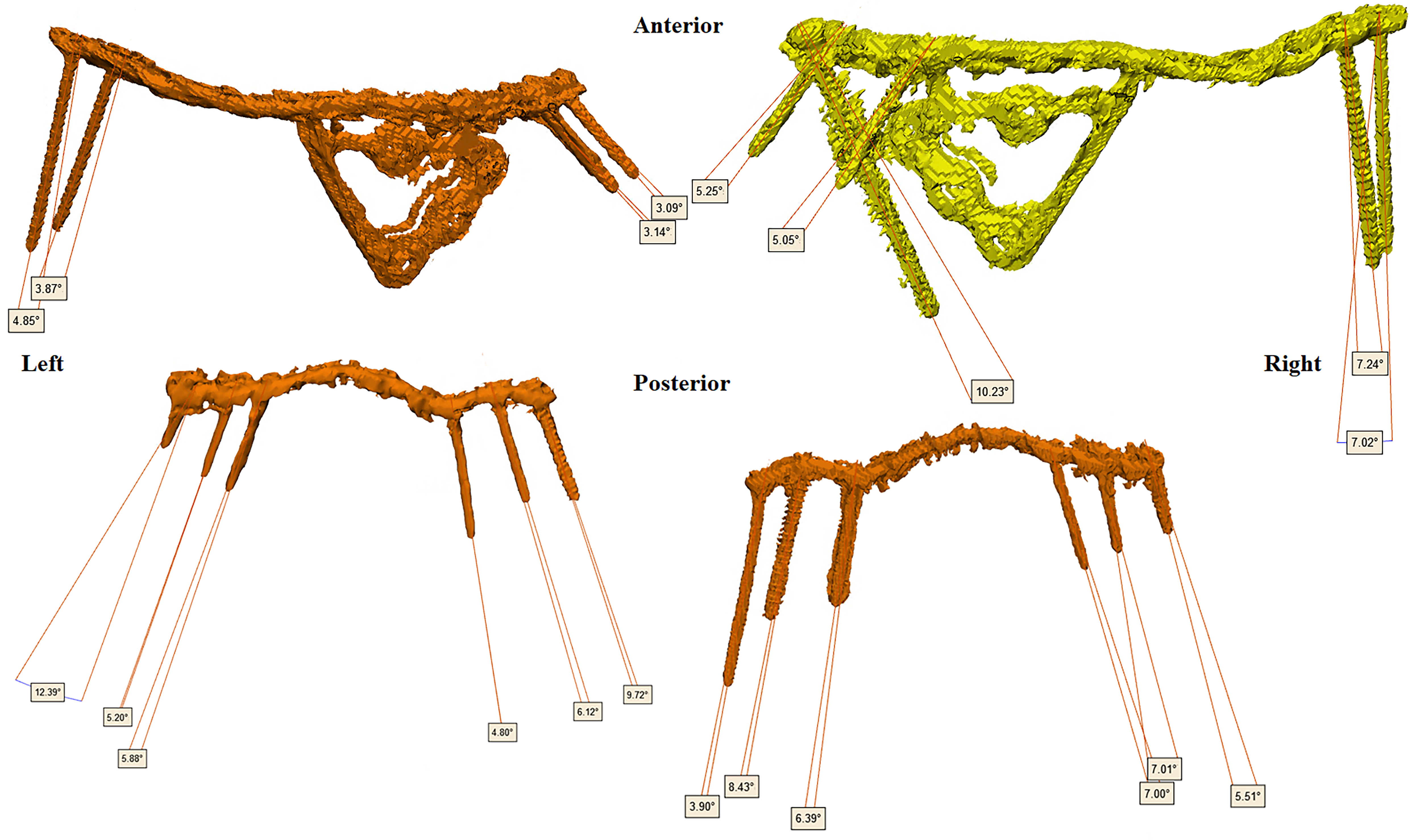

Supplement: Supplementary file 1 [file jpm-11-00763-s001.zip › Figure S3.png]

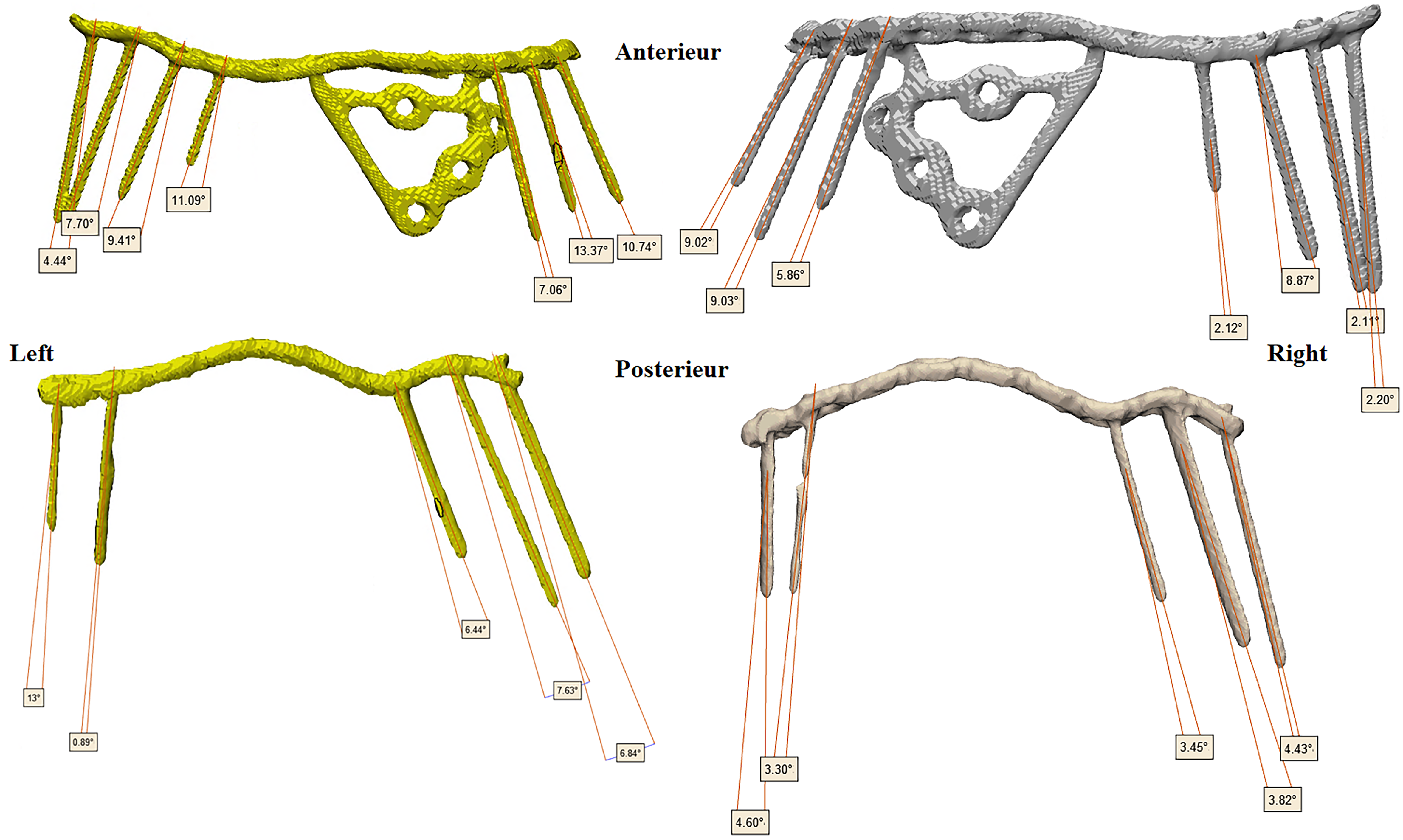

Supplement: Supplementary file 1 [file jpm-11-00763-s001.zip › Figure S4.png]
